# Violent and sexual victimisation and incident anxiety, mood and substance use disorders in childhood and adolescence: a co‐sibling study

**DOI:** 10.1111/jcpp.70144

**Published:** 2026-03-07

**Authors:** Joonas Pitkänen, Amir Sariaslan, Lauren Bishop, Mikko Aaltonen, Laura Mielityinen, Taina Laajasalo, Noora Ellonen, Pekka Martikainen

**Affiliations:** ^1^ Helsinki Institute for Demography and Population Health, Faculty of Social Sciences University of Helsinki Helsinki Finland; ^2^ The Max Planck – University of Helsinki Center for Social Inequalities in Population Health University of Helsinki Helsinki Finland; ^3^ Department of Psychiatry University of Oxford Oxford UK; ^4^ Law School University of Eastern Finland Joensuu Finland; ^5^ Faculty of Social Sciences Tampere University Tampere Finland; ^6^ Finnish Institute for Health and Welfare Helsinki Finland; ^7^ Max Planck Institute for Demographic Research Rostock Germany

**Keywords:** Violent victimisation, sexual victimisation, mood disorders, anxiety disorders, substance use disorders, sibling comparison

## Abstract

**Background:**

**S**tudies on the association between victimisation in childhood and adolescence and psychiatric disorders increasingly acknowledge that these associations might be partly confounded by unmeasured familial factors. However, previous quasi‐experimental evidence is largely based on retrospective self‐reported data with potential response biases and small samples.

**Methods:**

We measured psychiatric disorders and victimisation events from routinely collected administrative datasets on Finnish total birth cohorts 1996–2005. We identified all violent and sexual victimisation events using plaintiff information taken from registers containing data on crimes reported to the police between birth and the end of 2020. Incident anxiety, mood and substance use disorders were identified from registers containing records of inpatient and specialised outpatient psychiatric care. We compared all those exposed to victimisation to five population controls and their unexposed siblings, with the latter thereby adjusting for shared unobserved familial factors. We used stratified Cox regression models to estimate the associations between victimisation and the psychiatric disorders, with a follow‐up from victimisation until the outcome, exit from the population or the end of 2020.

**Results:**

Violent and sexual police‐reported victimisation were both associated with an increased risk of psychiatric disorders, with adjusted hazard ratios ranging between 2.3 (95% Confidence Interval [CI]: 2.2, 2.4) for the association between violent victimisation and mood disorders and 3.9 (3.7, 4.1) for the association between sexual victimisation and anxiety disorders. In the sibling comparisons, the associations attenuated but remained clearly elevated, with the corresponding hazard ratios ranging between 1.9 (1.7, 2.1) and 2.6 (2.3, 2.9).

**Conclusions:**

The results are consistent with a causal interpretation of the association between police‐reported victimisation and psychiatric disorders. Mental health‐related support after victimisation is an important task as it may prevent the onset of psychiatric disorders. Prevention of victimisation might decrease the number of psychiatric disorders in the population.

## Introduction

Early‐life exposure to violent and sexual victimisation is a well‐known risk factor for a wide range of common psychiatric disorders, including depression, anxiety and substance use (Baldwin et al., 2023; Burghart & Backhaus, 2024; Hailes, Yu, Danese, & Fazel, 2019; Li, D'Arcy, & Meng, 2016). Besides a causal pathway between victimisation and mental health, previous research has also suggested that the association might be partly due to confounding. Such confounding may stem from various sources. First, victimisation and psychiatric disorders share common risk factors. For instance, low parental socio‐economic position may expose children and adolescents to increased levels of family stress and neighbourhood deprivation, which could elevate the risk of both victimisation (Bywaters et al., 2016; Walsh, McCartney, Smith, & Armour, 2019) and psychiatric disorders (Reiss, 2013). Victimisation is also associated with other childhood adversities, which together are known to increase the risk of psychiatric disorders in a dose–response manner (Hughes et al., 2017).

Second, in addition to shared risk factors, some studies have suggested that the association between victimisation and psychiatric disorders might be, at least to some extent, genetically confounded (Baldwin et al., 2022, 2023; Dalvie et al., 2020; Johansson et al., 2022; Pittner et al., 2020; Ratanatharathorn et al., 2021; Warrier et al., 2021). Genetic confounding may arise through different pathways. Parental psychiatric disorders are associated with an elevated risk of child maltreatment (Stith et al., 2009) but most psychiatric disorders also show at least a modest degree of heritability (de Wit & Polderman, 2023), which induces a passive gene–environment correlation between child maltreatment and psychiatric disorders (Warrier et al., 2021). Additionally, a non‐passive gene–environment correlation between psychiatric disorders and victimisation occurs when the individual either seeks riskier environments or such environments react to the individual's behaviour (Warrier et al., 2021). For instance, individuals with substance use disorders have an elevated risk of spending time in environments characterised by violence. It is also known that individuals with psychiatric disorders have an elevated risk of being subjected to violence by the perpetrators (Sariaslan, Arseneault, Larsson, Lichtenstein, & Fazel, 2020). The presence of genetic confounding does not, however, imply genetic determinism nor that the victim is to blame. These processes only inform us about the complex nature of the association between exposure to victimisation and the heightened risk of psychiatric disorders at the population level.

Disentangling the underlying mechanisms and establishing the causal nature of the association between victimisation in childhood and adolescence and subsequent psychiatric disorders is of crucial importance in preventing both victimisation and psychiatric disorders (Baldwin et al., 2022, 2023; Warrier et al., 2021). A recent meta‐analysis (Baldwin et al., 2023) of family‐based studies and other quasi‐experimental evidence estimated that around 45% of the associations between indicators of child maltreatment and risks of psychiatric disorders was attributed to the quasi‐experimental adjustment, but a statistically significant effect of maltreatment on psychiatric disorders remained after adjustment. The indicators assessed in the meta‐analysis included physical, sexual and emotional abuse and neglect, institutional neglect and broader measures of adverse childhood experiences and victimisation. These findings were consistent with a related meta‐analysis on bullying victimisation (Schoeler, Duncan, Cecil, Ploubidis, & Pingault, 2018), with studies using genetic data to explain the associations with SNP heritability (Baldwin et al., 2022) or to conduct Mendelian randomisation (Warrier et al., 2021), and with studies using sibling comparisons to study victimisation occurring throughout the life course (Chen et al., 2024; Sariaslan et al., 2024).

However, important gaps in the current evidence from quasi‐experimental studies remain. First, previous studies that have been able to adjust for unmeasured familial confounders, such as twin‐ and sibling‐comparisons, have largely relied on retrospective self‐reports of victimisation (Baldwin et al., 2023). Previous research has shown that retrospective and prospective measurements of victimisation identify partly different populations (Baldwin, Reuben, Newbury, & Danese, 2019) and that self‐reported exposures might have stronger associations with psychiatric disorders than objective assessments (Danese & Widom, 2020). Second, only a few family‐based quasi‐experimental studies have examined differences between types of childhood victimisation (Barrigón et al., 2015; Bornovalova et al., 2012; Capusan et al., 2016; Daníelsdóttir et al., 2024; Kullberg et al., 2021; Magnusson et al., 2012). Third, many of the previous studies are based on relatively small samples. Finally, many of the family‐based studies have used exposures occurring only in the family environment, thus limiting variation between siblings (Schaefer et al., 2018).

In this study, we use Finnish total population data which contains routinely collected prospective information from the police and hospitals, with linkages between children and their family and household members. We establish a quasi‐experimentally adjusted estimate of the association between exposure to police‐reported violent and sexual victimisation and the incidence of hospital‐treated anxiety, mood and substance use disorders in childhood and adolescence by comparing differences in the diagnoses between the exposed individuals and their unexposed siblings. Our main analysis focuses on broad measures of victimisation, but we also provide additional details on the perpetrators of the events.

## Methods

### Data

The study is based on register data obtained from Statistics Finland on all individuals born in Finland between 1996 and 2005 with information on both biological parents at birth (*n* = 560,316). For all individuals and their parents, we obtained information on sociodemographic characteristics and police‐reported crimes from Statistics Finland, which were linked with data on inpatient and outpatient hospital care from the Finnish Institute for Health and Welfare. The oldest cohort was chosen based on the availability of the police‐reported crime data (1996 onwards); the youngest was selected as they had turned 15 by the end of follow‐up, an age by which we could reasonably expect to observe a sufficient onset of the studied outcomes (Solmi et al., 2021). The use of the data has been approved by the Statistics Finland's Board of Statistical Ethics (permission number TK/2575/07.03.00/2025) and Findata (permission number THL/5298/14.06.00/2025). According to Finnish legislation, informed consent is not required in studies using only register data. We followed the STROBE reporting guidelines (Table S1).

### Police‐reported victimisation and study design

Using data on police‐reported crime, plaintiff ids and dates of crime, we identified the first exposures of victimisations to non‐lethal violent and sexual crimes between birth and the end of 2020 for each individual. See Table 1 for the included crimes. Individuals who had previously been treated for the studied outcomes (4,707 with violent and 1,729 with sexual victimisation), or who exited before or at the index date were not included. The latter exclusions (290 with violent and 87 with sexual victimisation) were mostly individuals who emigrated before victimisation but later reappeared in the data. After these exclusions, the analytic population comprised 25,364 individuals with records of violent victimisation and 6,915 individuals with records of sexual victimisation.

**Table 1:** Definitions and data sources of the variables used

Variable	Definition	Data source
Violent victimisation	Victimisation defined as being a plaintiff in a police‐report between birth and 31 December 2020.^a^ Crimes included: minor assault, assault, aggravated assault, attempted homicide. First victimisation chosen as the index event, based on the dates of the police reports.^b^ The data only includes crimes where circumstances, parties involved and other elements necessary for deciding on charges have been clarified by the police.	Statistics Finland, police‐reported crime (1996–2020)
Sexual victimisation	Victimisation defined as being a plaintiff in a police‐report between birth and 31 December 2020.^a^ Following crimes included: child sexual abuse, rape and other sexual offences. First victimisation chosen as the index event, based on the dates of the police reports.^b^ The data only includes crimes where circumstances, parties involved and other elements necessary for deciding on charges have been clarified by the police.	Statistics Finland, police‐reported crime (1996–2020)
Substance use disorders	The child had an inpatient episode or outpatient visit in hospital‐level facilities with ICD‐10 diagnoses F10–F19. Primary and secondary diagnoses included. Date of first event used as the outcome date.	Finnish Institute for Health and Welfare (inpatient 1970–2020, outpatient 1998–2020)
Mood disorders	The child had an inpatient episode or outpatient visit in hospital‐level facilities with ICD‐10 diagnoses F30–F39. Primary and secondary diagnoses included. Date of first event used as the outcome date.	Finnish Institute for Health and Welfare (inpatient 1970–2020, outpatient 1998–2020)
Anxiety disorders	The child had an inpatient episode or outpatient visit in hospital‐level facilities with ICD‐ 10 diagnoses F40–F48. Primary and secondary diagnoses included. Date of first event used as the outcome date.	Finnish Institute for Health and Welfare (inpatient 1970–2020, outpatient 1998–2020)
Two‐parent family	An indicator for whether the child resided with in a two‐parent family at birth.	Statistics Finland (1987–2020)
Parental education	Highest education of biological parents at childbirth, classified into no secondary education (ISCED 0–2), upper secondary education (ISCED 3–4) and any tertiary education (ISCED 5–8)	Statistics Finland (1987–2020)
Foreign background	Either biological parent was born abroad.	Statistics Finland (1987–2020)
Parental social assistance	Either biological parent received at least one euro of means‐tested social assistance during the year the child was born.	Statistics Finland (1987–2020)
Parental psychiatric disorder	Either biological parent had an inpatient episode or outpatient visit to a hospital‐level facility with any psychiatric diagnosis (ICD8: 290–315; ICD9: 290–319; ICD10: F00–F99) before the child was born.	Finnish Institute for Health and Welfare (inpatient 1970–2020, outpatient 1998–2020)
Parental crime	Either biological parent was convicted for any violent or sexual crime before the child was born.	Statistics Finland, criminal conviction data (1977–2020)
Sex	Latest information on person's sex, categorised as men and women.	Statistics Finland (1987–2020)
Region of residence at birth	Region of residence at birth in NUTS2^c^ categories [Helsinki‐Uusimaa, Western Finland (incl. Åland), Southern Finland, and Eastern and Northern Finland]. Used only in the population‐level analysis as a matching identifier and a strata variable.	Statistics Finland (1987–2020)
Birth year	Person's year of birth. Used in the population‐level analysis as a matching identifier and a strata variable in 1‐year categories. Used as a control variable in the sibling comparison, recategorised into 1996–2000 and 2001–2005.	Statistics Finland (1987–2020)
Birth month	Person's month of birth (1996–2005). Used in the population‐level analysis only, as a matching identifier and a strata variable in 1‐month categories.	Statistics Finland (1987–2020)

^a^ In some cases, plaintiffs can be also individuals other than victims of the crime but this is rare at the ages studied here.

^b^ If the date of offence was missing, the date of the police report was used.

^c^ Nomenclature of territorial units for statistics.

We then used exposure density sampling (EDS) (Chen et al., 2024; Ohneberg, Beyersmann, & Schumacher, 2019) to match five as‐yet‐unexposed population controls to each case from the total population. We used EDS to avoid conditioning on future exposures, which might yield biased results (Ohneberg et al., 2019). Individuals with events who were excluded due to previous treatment of the studied outcomes or exits before victimisation were included in the total population from which the controls were sampled. Hence, these individuals could act as controls until they experienced the outcome or exited the population. Matching was conducted separately for the violent and sexual victimisation events, by sex, month and year of birth, and region of residence at birth.

To assess familial confounding, we compared each individual with a victimisation event to their full biological siblings in the studied birth cohorts who were as‐yet‐unexposed to either victimisation or the studied outcomes. Due to the age‐graded presentation of both victimisation and psychiatric disorders, we used attained age as the underlying time scale to identify the unexposed sibling controls. These comparisons comprised 11,175 individuals exposed to violent victimisation and their 14,003 as‐yet‐unexposed siblings nested in 10,494 sibships and 3,423 individuals exposed to sexual victimisation and their 4,673 as‐yet‐unexposed siblings nested in 3,272 sibships. See Figure S1 for further details of the study population formation.

For descriptive purposes, we calculated the number of subsequent victimisations after the initial event for each included case. We also calculated the number of cases with victimisation who had any records of violent or sexual offending between birth and the end of 2020. We then identified the perpetrators of the index events using perpetrator identifiers and the available family and household linkages. Due to small sample sizes, particularly in the sibling comparisons, these variables were not included in the regression analyses. Similarly, we were not able to conduct subgroup analyses by different demographic characteristics.

### Outcome variables

We identified incident hospital‐presenting mental and behavioural disorders due to psychoactive substance use, mood disorders, and anxiety and stress‐related disorders from data on inpatient and outpatient hospital care obtained from the Finnish Institute for Health and Welfare (ICD‐10 codes in Table 1).

### Statistical modelling

In the population comparisons, we used Cox models with attained age as the underlying time scale (i.e. each individual entered at the age of exposure or sampling and exited at the age of the outcome or censoring). All individuals were followed until the date of hospital‐presenting psychiatric disorder, death, emigration or the end of 2020. In the EDS approach, each individual with an event is matched to as‐yet‐unexposed controls who are sampled from individuals who are free of the exposure and at risk of the outcome at the specific event date (Ohneberg et al., 2019).

From this approach of matching as‐yet‐unexposed controls to cases, it follows that cases can act as controls before their date of exposure. For these individuals, the date of first sampling was considered the entry date and exposure to victimisation was added into the models as a time‐varying variable. Furthermore, individuals can be sampled as controls for multiple cases if they remain as‐yet‐unexposed to the exposure and outcome before the specific index date. For these controls, the date of first sampling was considered the entry date. A tutorial on how to perform EDS using R and a schematic example of the approach can be found in Ohneberg et al., 2019.

For the individuals exposed to victimisation and their matched population controls, we fitted a crude model and a covariate‐adjusted model. The following covariates, derived from data provided by Statistics Finland, were measured at birth to have comparable measures regardless of age of exposure to victimisation: two‐parent family, parental education, foreign background and parental means‐tested social assistance. In addition, we included a covariate for any hospital‐presenting parental psychiatric disorder before the child's birth, measured using data from the Finnish Institute for Health and Welfare, and a covariate for any parental conviction of a violent or sexual crime before the child's birth, measured using data on criminal convictions from Statistics Finland. In both models, we stratified by the variables used in matching (sex, month and year of birth and region of residence at birth). See Table 1 for further details on all variables used in the analyses.

In the sibling comparisons, we again used attained age as the underlying time scale. The unexposed siblings were followed from the date they were exactly the same age as the exposed siblings were at the time of their exposure, and follow‐up ended at the date of hospital‐presenting psychiatric disorder, death, emigration or the end of 2020. We estimated Cox regression models stratified by sibship and adjusted for sex, birth year, parental social assistance receipt and two‐parent family status at birth. Other covariates were not included due to minimal variation between siblings. The exposed individuals were entered into the models with all their unexposed siblings. In sibships where multiple siblings were exposed to victimisation, we added a separate stratum for each exposed individual and their as‐yet‐unexposed sibling controls. The sibling controls who were later exposed to victimisation were censored at the date of victimisation. Hence, individuals with victimisation could act as controls for other siblings in the sibship before their own event. As the same persons could enter the model multiple times in separate strata, we clustered the standard errors of the model on the individual.

### Additional analyses

To assess whether the inclusion of stress‐related disorders into anxiety disorders was driving the results, we conducted an additional analysis excluding these disorders from the events. Individuals who only presented with stress‐related disorders were censored at the event date.

## Results

### Descriptive results

The median age of violent victimisation was 15 in the population cohort and 14 in the sibling cohort; the median age of sexual victimisation was 14 in both cohorts (Table 2). The median follow‐up times ranged between 5 and 7 years, depending on the cohort, and were generally slightly longer among unexposed individuals, except regarding violent victimisation in the sibling cohort and sexual victimisation and substance use in the same cohort. The median ages of the outcomes ranged between 16 and 18 for anxiety and mood disorders, and between 17 and 19 for substance use. The rates of psychiatric disorders were around 3–5 times higher among those exposed to victimisation compared to the matched population controls, and around 1.5–3.5 times higher among the siblings exposed to victimisation compared to unexposed siblings (Table 2). Social disadvantage was more common among those exposed to victimisation than among the population controls. Having a parent with a foreign background was slightly more common among the exposed compared to the matched controls. Men were overrepresented in the violent victimisation cohort (70%), whereas women comprised the majority of the sexual victimisation cohort (87%).

**Table 2:** Descriptive characteristics of the study populations

	Violent victimisation				Sexual victimisation			
	Population analysis		Sibling comparison		Population analysis		Sibling comparison	
	Exposed	Unexposed	Exposed	Unexposed	Exposed	Unexposed	Exposed	Unexposed
	*N* = 25,364	*N* = 126,820	*N* = 11,175	*N* = 14,996	*N* = 6,915	*N* = 34,575	*N* = 3,423	*N* = 4,904
Median age at index date (IQR)	15 (11,18)	15 (11,18)	14 (11,17)	14 (11,17)	14 (10,15)	14 (10,15)	14 (10,15)	14 (10,15)
Anxiety disorders								
*N* with an event (Rate/1000 person‐years)	3,656 (24.2)	6,030 (8.3)	1,514 (21.5)	1,137 (13.8)	2,310 (53.4)	3,057 (12.4)	1,112 (51.4)	491 (14.6)
Median follow‐up time	5.2	5.9	5.6	4.8	5.4	7.0	5.4	6.2
Median age at outcome (IQR)	17 (15,20)	18 (15,20)	17 (15,19)	17 (15,20)	16 (15,18)	17 (15,19)	16 (15,18)	17 (15,19)
Mood disorders								
*N* with an event (Rate/1000 person‐years)	3,026 (19.6)	5,366 (7.4)	1,315 (18.4)	1,019 (12.3)	1,757 (37.8)	2,732 (11.0)	855 (37.2)	466 (13.8)
Median follow‐up time	5.3	5.9	5.7	4.8	6.1	7.1	6.1	6.2
Median age at outcome (IQR)	17 (15,20)	18 (16,20)	17 (15,19)	17 (15,19)	16 (15,18)	17 (16,19)	16 (15,18)	17 (15,19)
Substance use disorders								
*N* with an event (Rate/1000 person‐years)	2,031 (12.7)	2,008 (2.7)	842 (11.4)	411 (4.8)	611 (11.7)	664 (2.6)	294 (11.4)	184 (5.3)
Median follow‐up time in years	5.5	6.0	6.0	4.9	7.1	7.4	7.0	6.4
Median age at outcome (IQR)	19 (16,20)	19 (17,21)	18 (16,20)	18 (16,20)	18 (16,19)	19 (16,20)	17 (16,19)	18 (17,20)
Two‐parent family at birth, *N* (%)								
No	3,738 (15)	8,400 (7)	1,072 (10)	1,383 (9)	974 (14)	2,380 (7)	301 (9)	376 (8)
Yes	21,626 (85)	118,420 (93)	10,103 (90)	13,613 (91)	5,941 (86)	32,195 (93)	3,122 (91)	4,528 (92)
Parental education at birth, *N* (%)								
Basic education	3,932 (16)	7,331 (6)	1,515 (14)	2,116 (14)	1,046 (15)	2,153 (6)	454 (13)	629 (13)
Secondary education	12,755 (50)	49,499 (39)	5,424 (49)	7,366 (49)	3,603 (52)	13,650 (39)	1,742 (51)	2,527 (52)
Tertiary education	8,677 (34)	69,990 (55)	4,236 (38)	5,514 (37)	2,266 (33)	18,772 (54)	1,227 (36)	1,748 (36)
Foreign background, *N* (%)								
No	22,001 (87)	116,903 (92)	9,680 (87)	12,797 (85)	6,247 (90)	31,820 (92)	3,099 (91)	4,441 (91)
Yes	3,363 (13)	9,917 (8)	1,495 (13)	2,199 (15)	668 (10)	2,755 (8)	324 (9)	463 (9)
Parental social assistance at birth, *N* (%)								
No	16,645 (66)	107,896 (85)	7,810 (70)	10,426 (70)	4,530 (66)	29,281 (85)	2,392 (70)	3,475 (71)
Yes	8,719 (34)	18,924 (15)	3,365 (30)	4,570 (30)	2,385 (34)	5,294 (15)	1,031 (30)	1,429 (29)
Parental psychiatric disorder, *N* (%)								
No	20,905 (82)	115,804 (91)	9,513 (85)	12,838 (86)	5,567 (81)	3,1,437 (91)	2,869 (84)	4,150 (85)
Yes	4,459 (18)	11,016 (9)	1,662 (15)	2,158 (14)	1,348 (19)	3,138 (9)	554 (16)	754 (15)
Parental crime, *N* (%)								
No	20,655 (81)	116,897 (92)	9,365 (84)	12,627 (84)	5,739 (83)	31,735 (92)	2,942 (86)	4,270 (87)
Yes	4,709 (19)	9,923 (8)	1,810 (16)	2,369 (16)	1,176 (17)	2,840 (8)	481 (14)	634 (13)
Child's sex, *N* (%)								
Men	17,169 (68)	85,845 (68)	7,826 (70)	7,533 (50)	885 (13)	4,425 (13)	437 (13)	2,662 (54)
Women	8,195 (32)	40,975 (32)	3,349 (30)	7,463 (50)	6,030 (87)	30,150 (87)	2,986 (87)	2,242 (46)

Ages at exposure and outcome measured as exact ages and rounded to closest age.

Over half of the violent victimisations were perpetrated by non‐household members who were of a similar age (maximum 10 years older) to the person exposed to victimisation (Table 3). Conversely, most sexual victimisation events (62%) were perpetrated by older (>10 years older) non‐household members. Twenty‐eight per cent of those exposed to violent victimisation and 10% of those exposed to sexual victimisation also had records of being perpetrators in violent offences, but very few individuals had a record of being a perpetrator in a sexual offence. A higher proportion of those exposed to sexual victimisation were also exposed to violent victimisation than vice versa. Finally, subsequent violent victimisation events were more common than subsequent events of sexual victimisation.

**Table 3:** Frequencies (%) of perpetrator characteristics, subsequent victimisation, violent and sexual offending and both types of victimisations among those with a police‐report of violent or sexual victimisation

Perpetrator^a^ characteristics	Violent victimisation	Sexual victimisation
Family or household member,^b^ *N* (%)	6,534 (26)	1,018 (15)
Non‐household members, ≤10 years older than the plaintiff, *N* (%)	12,858 (51)	1,634 (24)
Non‐household members, >10 years older than the plaintiff,^c^ *N* (%)	5,972 (24)	4,263 (62)
Subsequent victimisations of the same type		
No, *N* (%)	20,433 (81)	5,983 (87)
Yes, *N* (%)	4,931 (19)	932 (13)
Any violent offending in data		
No, *N* (%)	18,313 (72)	6,252 (90)
Yes, *N* (%)	7,051 (28)	663 (10)
Any sexual offending in data		
No, *N* (%)	24,881 (98)	6,865 (99)
Yes, *N* (%)	483 (2)	50 (1)
Both victimisations		
No, *N* (%)	24,146 (95)	5,697 (82)
Yes, *N* (%)	1,218 (5)	1,218 (18)

^a^ In the case of multiple perpetrators, priority was given to household members, followed by younger perpetrators.

^b^ Includes non‐resident and co‐resident biological parents, full and half‐siblings, biological parent's partners as registered in residential unions at the end of the year preceding the index event, children of the biological parents' partners and any other individuals residing in the same household at the end of the year preceding victimisation.

^c^ Includes events where the perpetrator is missing (439 violent and 199 sexual victimisations).

### Regression models

**Figure 1:**
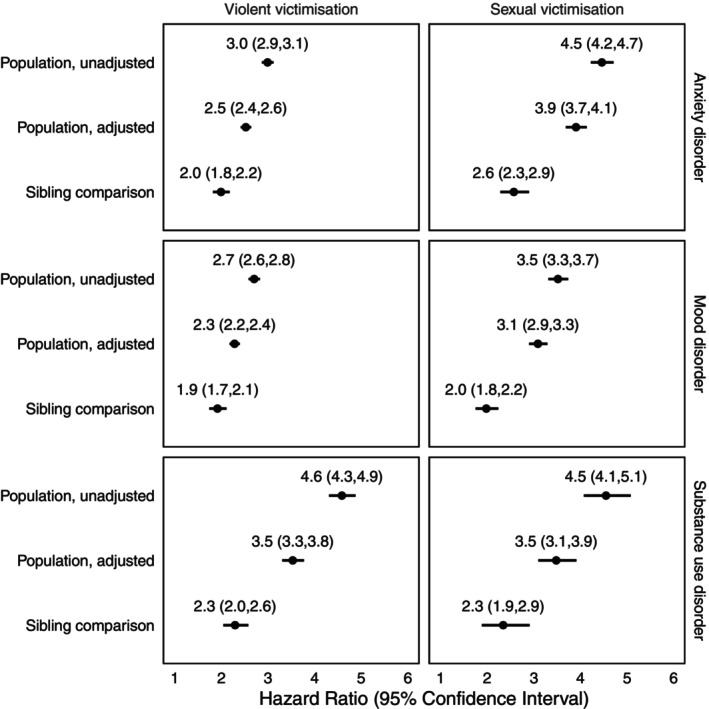
The associations between police‐report of violent and sexual victimisation and anxiety, mood and substance use disorders. The population, unadjusted models compare those exposed to victimisation with their five matched population controls (matched by sex, birth year, birth month and region of residence). The models are stratified by the matching variables. The population, adjusted models are further adjusted for two‐parent family, parental education, foreign background and parental means‐tested social assistance at birth and parental psychiatric disorders and violent and sexual crime convictions before birth. The sibling comparison models compare those exposed to victimisation with their unexposed siblings. The models are stratified by the sibling identifier and adjusted for sex, birth year, parental social assistance receipt and two‐parent family at birth

Violent victimisation was associated with around three times higher hazard rates for anxiety and mood disorders (HR = 3.0, 95% CI: 2.9, 3.1 and HR = 2.7, 95% CI: 2.6, 2.8, respectively), and almost five times higher hazard rates for substance use disorders (HR = 4.6, 95% CI: 4.3, 4.9) in the unadjusted models compared to the matched population controls (Figure 1). After adjustment for measured confounders, these estimates were between 16% and 23% lower, with HRs of 2.5 (2.4, 2.6) for anxiety disorders, 2.3 (2.2, 2.4) for mood disorders and 3.5 (3.3, 3.8) for substance use disorders. In the sibling comparisons, the estimates were further attenuated by 21% for anxiety disorders (HR = 2.0, 95% CI: 1.8, 2.2), 16% for mood disorders (HR = 1.9, 95% CI: 1.7, 2.1) and 35% for substance use disorders (HR = 2.3, 95% CI: 1.9, 2.9; Figure 1). These HRs should be interpreted as weighted averages over the whole follow‐up, as results from the Schoenfeld residual tests indicated a violation of the proportional hazards assumption in many of the models at earlier ages (Figures S2 and S3), when the outcomes are rather rare (Table 2).

Sexual victimisation was associated with more than fourfold increased hazards for anxiety (HR = 4.5, 95% CI: 4.3, 4.7) and substance use disorders (HR = 4.5, 95% CI: 4.1, 5.1) in the unadjusted models with the matched population controls, and 3.5 times higher hazards for mood disorders (HR = 3.5, 95% CI: 3.3, 3.7). The estimates attenuated by 12% and 23% after covariate adjustment for anxiety (HR = 3.9, 95% CI: 3.7, 4.1) and mood disorders (HR = 3.1, 95% CI: 2.9, 3.3) and substance use disorders (HR = 3.5, 95% CI: 3.1, 3.9), respectively. All the estimates further attenuated by around one third after adjustment for unobserved confounding in the sibling comparisons, with HRs of 2.6 (2.3, 2.9) for anxiety disorders, 2.0 (1.8, 2.2) for mood disorders and 2.3 (1.9, 2.9) for substance use disorders.

Removing stress‐related disorders from the anxiety disorders outcome dampened the association between both types of victimisation and anxiety disorders, especially for sexual victimisation, but the risk of the outcome remained clearly elevated in both the population and the sibling comparison. The adjusted HRs in the population comparison were 2.1 (95% CI: 2.0, 2.2) for violent and 2.7 (2.5, 2.9) for sexual victimisation, and 1.7 (1.6, 1.9) and 1.8 (1.6, 2.1) in the respective sibling comparisons (Figure S4).

## Discussion

We conducted a co‐sibling study on the association between exposure to violent or sexual victimisation and subsequent hospital‐treated mood, anxiety and substance use disorders. Our results show that after accounting for familial factors shared between siblings as well as observed confounders not shared by siblings at birth, estimates of the associations between exposures and victimisation were between 30% and 50% lower than the respective unadjusted associations in the population‐level analyses. Nevertheless, the associations between exposures to both types of victimisation and psychiatric disorders were robust, and the relative hazards for psychiatric disorders were around two times higher when the victims were compared to their unexposed siblings. The effect sizes in the sibling comparisons were similar regardless of the type of victimisation. While not definitively proving causality, these within‐family associations are consistent with a causal interpretation of the association between victimisation and psychiatric disorders.

The magnitude of the within‐sibship associations between exposure to victimisation and psychiatric disorders in childhood and adolescence observed in this study (HRs ranging between 1.9 and 2.6) were similar to previous studies using Nordic administrative registers to study exposure to victimisation throughout the life course (Chen et al., 2024; Sariaslan et al., 2024) and in childhood specifically (Capusan et al., 2021). All these studies reported within‐sibship HRs ranging approximately between 1.7 and 3.1 for the association between victimisation and the outcomes comparable to those used in this study.

Our findings are also consistent in direction with previous meta‐analyses, which have reported Cohen's *d* values of 0.31 for the association between child maltreatment and mental health problems (Baldwin et al., 2023) and 0.27 and 0.15 for the associations between bullying and internalising and externalising outcomes, respectively (Schoeler et al., 2018). Both meta‐analyses categorise these as small effects based on the Cohen's *d* cut points of 0.2 for small, 0.5 for moderate and 0.8 for large effects. An approximation of Cohen's *d*, assuming that the hazard ratio equals the odds ratio and using the scaling factor of 1.81 from Chinn (2000), would translate our hazard ratios into effect sizes ranging between 0.35 and 0.53. The lowest value is close to Baldwin et al. (2023) and is between a small and moderate effect size, and the highest corresponds to moderate effect size. Our somewhat higher effect sizes compared to the meta‐analytic estimates might be related to the severity of our exposures and outcomes. The victimisation events were reported to police and all the psychiatric outcomes required contact with specialised healthcare services. These observed differences may also relate to other study characteristics, such as exposure and outcome definitions, sample sizes and other methodological choices. In addition, the Finnish welfare state is a rather unique context characterised by a universal healthcare system and a generally high public trust in the police and authorities, which could add context‐dependency to our results.

Furthermore, the types of the recorded events might be different from studies that focus on, for example, child maltreatment, which is commonly defined as victimisation by family members. In our study, the violent exposures were most commonly perpetrated by similarly aged peers, followed by exposures perpetrated by family members and non‐household members substantially older than the victim. Of the sexual victimisation cases, the vast majority were perpetrated by older non‐household members. Due to small sample sizes especially in the sibling comparison, we were not able to examine whether the associations between victimisation and the outcomes differ by perpetrator characteristics but recommend that future research with a quasi‐experimental design delve deeper into such associations. Either way, our results do imply that in the development of preventive and supportive measures, focusing on broader measures of victimisation in addition to child maltreatment occurring in the family environment might be useful. Similarly, further research on multiple victimisations, poly‐victimisation and the victim‐offender overlap may also provide important insights into the total association examined in this study. A very important omission from this study and a crucial topic for a future study would be to test whether gender moderates these quasi‐experimentally adjusted associations. We were not able to robustly conduct the sibling comparisons stratified by the child's sex due to very few men exposed to sexual victimisation on the one hand, and few women experiencing substance use disorders on the other. Such differences deserve further attention.

In the interpretation of the findings, several methodological issues need to be considered. First, we used routinely collected data from the police and hospital‐level facilities to establish both victimisation events and outcomes. Hence, unreported victimisation events are not included in our study, nor are psychiatric disorders that remain untreated or are treated in primary care. On the other hand, both the victimisation events and the psychiatric outcomes are objectively assessed and based on prospective data, and thus likely to be true positives. Second, although we had exact dates of victimisation and hospital visits, these could differ from the actual onsets of both the exposures and the outcomes. Third, our sibling comparisons rely on information from discordantly exposed siblings. However, it is possible that the unexposed siblings may have been exposed to violence not reported to the police. The siblings might also have directly witnessed the violence towards their sibling, which might constitute a traumatic experience. Some of the attenuation in the sibling analyses might also be due to inflation of measurement error in fixed effects designs (Frisell, Öberg, Kuja‐Halkola, & Sjölander, 2012). However, these biases would most likely yield overly conservative rather than exaggerated estimates. Finally, even though sibling comparison designs are relatively strong, it is possible that some of the association is still confounded by, for example, genetic factors or early life peer effects not shared by the siblings.

Overall, our results on approximately doubled hazards for psychiatric disorders in the within‐family comparisons imply that enhancing efforts to prevent victimisation might also contribute to reduced psychiatric disorders at the population level (Baldwin et al., 2023). Even if such reductions would be small, there is a strong moral obligation to protect children and adolescents from physical and sexual violence, bullying and other types of victimisation. Victimised individuals also need specialised clinical services to reduce the possible mental health consequences of victimisation, taking into account both the traumatic experience and other risk factors of psychiatric disorders (Baldwin et al., 2023). Given potential spillover effects, support services might also benefit the siblings and other family members of those exposed to victimisation. Building individual and community resilience to adverse experiences might be useful (Longhi, Brown, & Fromm Reed, 2021), as it seems unlikely that all interpersonal violence could be eradicated. Furthermore, the findings also suggest that collaborations between the police and psychiatric healthcare might be useful, in terms of mapping the needs of those exposed to victimisation and establishing relevant treatment contacts after victimisation has been reported to the police. Finally, individuals with previous psychiatric disorders have a higher risk of victimisation (Sariaslan et al., 2020), and, as shown in this study and elsewhere, previously victimised individuals have a high risk of re‐victimisation (Goemans, Viding, & McCrory, 2023). This suggests that preventing victimisation among these populations is also of importance.

## Ethical considerations

According to Finnish legislation, informed consent is not required in studies using only register data. The use of the data has been approved by the Statistics Finland's Board of Statistical Ethics (date of approval 19 November 2025, approval number TK/2575/07.03.00/2025) and Findata (date of approval 14 November 2025, approval number THL/5298/14.06.00/2025).

**Key points**

**What's known**

- The association between early‐life violent and sexual victimisation and psychiatric disorders is well established, but existing evidence mostly comes from observational studies prone to potential bias due to unobserved familial confounding.

**What's new**

- This study used Finnish register data and identified around 20,000 violent victimisations and 7,000 sexual victimisations in childhood and adolescence. Incident anxiety, mood and substance use disorders were studied as outcomes, and sibling comparisons were used to adjust for familial confounding.
- Compared to population level associations, the estimates adjusting for familial confounding attenuated but remained clearly elevated, with hazard ratios ranging between 1.9 and 2.6.

**What's relevant**

- Efforts to prevent violent and sexual victimisation and to support the psychiatric well‐being of victimised individuals remain important public health tasks.

## Supporting information


**Table S1.** STROBE checklist.
**Figure S1.** Flowchart of the study population formation.
**Figure S2.** Results from the Schoenfeld residuals tests in the cohort comparisons.
**Figure S3.** Results from the Schoenfeld residuals tests in the sibling comparisons.
**Figure S4.** Results from a sensitivity analysis excluding stress‐related disorders from anxiety disorders.

## Data Availability

The underlying data in this work cannot be shared publicly. Permissions to use the data can be sought from Statistics Finland and Findata.
