# Supplementary material for: Violent and sexual victimisation and incident anxiety, mood and substance use disorders in childhood and adolescence: a co‐sibling study
Source: J Child Psychol Psychiatry. 2026 Mar 7;67(8):1383–92. doi: 10.1111/jcpp.70144 (PMC13341397; doi:10.1111/jcpp.70144)

**Violent and sexual victimisation and incident anxiety, mood and substance use disorders in childhood and adolescence: a co-sibling study**

**Supporting Information**

Table S1: STROBE checklist

Figure S1: Flowchart of the study population formation

Figure S2: Results from the Schoenfeld residuals tests in the cohort comparisons

Figure S3: Results from the Schoenfeld residuals tests in the sibling comparisons

Figure S4: Results from a sensitivity analysis excluding stress-related disorders from anxiety disorders

Table S1: STROBE Checklist

|  | Item | Recommendation | Relevant part of manuscript |  |  |
| --- | --- | --- | --- | --- | --- |
| **Title and abstract** | 1 | (*a*) Indicate the study’s design with a commonly used term in the title or the abstract | Title and abstract |  |  |
|  |  | (*b*) Provide in the abstract an informative and balanced summary of what was done and what was found | Title and abstract |  |  |
| Introduction | | |  |  |  |
| Background/rationale | 2 | Explain the scientific background and rationale for the investigation being reported | Introduction, paragraphs 1–3 |  |  |
| Objectives | 3 | State specific objectives, including any prespecified hypotheses | Introduction, paragraph 4 |  |  |
| Methods | | |  |  |  |
| Study design | 4 | Present key elements of study design early in the paper | Abstract, Methods |  |  |
| Setting | 5 | Describe the setting, locations, and relevant dates, including periods of recruitment, exposure, follow-up, and data collection | Methods, paragraphs 1–6 |  |  |
| Participants | 6 | (*a*) Give the eligibility criteria, and the sources and methods of selection of participants. Describe methods of follow-up | Methods, paragraphs 1–6 |  |  |
|  |  | (*b*) For matched studies, give matching criteria and number of exposed and unexposed | Methods, paragraph 2 |  |  |
| Variables | 7 | Clearly define all outcomes, exposures, predictors, potential confounders, and effect modifiers. Give diagnostic criteria, if applicable | Table 1 |  |  |
| Data sources/ measurement | 8* | For each variable of interest, give sources of data and details of methods of assessment (measurement). Describe comparability of assessment methods if there is more than one group | Table 1 |  |  |
| Bias | 9 | Describe any efforts to address potential sources of bias | Methods, paragraph 8; Discussion, paragraph 4 |  |  |
| Study size | 10 | Explain how the study size was arrived at | Supplementary Figure 1 |  |  |
| Quantitative variables | 11 | Explain how quantitative variables were handled in the analyses. If applicable, describe which groupings were chosen and why | Methods, paragraphs 2–7; Table 1 |  |  |
| Statistical methods | 12 | (*a*) Describe all statistical methods, including those used to control for confounding | Methods, paragraphs 2–7; Table 1 |  |  |
|  |  | (*b*) Describe any methods used to examine subgroups and interactions | Methods, paragraph 8 |  |  |
|  |  | (*c*) Explain how missing data were addressed | NA |  |  |
|  |  | (*d*) If applicable, explain how loss to follow-up was addressed | NA |  |  |
|  |  | (*e*) Describe any sensitivity analyses | Methods, paragraph 8 |  |  |
| Results | | |  |  |  |
| Participants | 13* | (a) Report numbers of individuals at each stage of study—eg numbers potentially eligible, examined for eligibility, confirmed eligible, included in the study, completing follow-up, and analysed | Supplementary Figure 1 |  |  |
|  |  | (b) Give reasons for non-participation at each stage | NA |  |  |
|  |  | (c) Consider use of a flow diagram | Supplementary Figure 1 |  |  |
| Descriptive data | 14* | (a) Give characteristics of study participants (eg demographic, clinical, social) and information on exposures and potential confounders | Table 2 |  |  |
|  |  | (b) Indicate number of participants with missing data for each variable of interest | NA |  |  |
|  |  | (c) Summarise follow-up time (eg, average and total amount) | Table 2 |  |  |
| Outcome data | 15* | Report numbers of outcome events or summary measures over time | Table 2 |  |  |
| Main results | 16 | (*a*) Give unadjusted estimates and, if applicable, confounder-adjusted estimates and their precision (eg, 95% confidence interval). Make clear which confounders were adjusted for and why they were included | Figure 1; Results, paragraphs 1–3 |  |  |
|  |  | (*b*) Report category boundaries when continuous variables were categorized | NA |  |  |
|  |  | (*c*) If relevant, consider translating estimates of relative risk into absolute risk for a meaningful time period | Not conducted |  |  |
| Other analyses | 17 | Report other analyses done—eg analyses of subgroups and interactions, and sensitivity analyses | Results, paragraphs 4–5 |  |  |
| Discussion | | |  |  |  |
| Key results | 18 | Summarise key results with reference to study objectives | Discussion, paragraph 1 |  |  |
| Limitations | 19 | Discuss limitations of the study, taking into account sources of potential bias or imprecision. Discuss both direction and magnitude of any potential bias | Discussion, paragraph 4 |  |  |
| Interpretation | 20 | Give a cautious overall interpretation of results considering objectives, limitations, multiplicity of analyses, results from similar studies, and other relevant evidence | Discussion, paragraphs 1–3, 5 |  |  |
| Generalisability | 21 | Discuss the generalisability (external validity) of the study results | Discussion, paragraph 2 |  |  |
| Other information | | |  |  |  |
| Funding | 22 | Give the source of funding and the role of the funders for the present study and, if applicable, for the original study on which the present article is based | Funding |  |  |

Figure S1: Flowchart of the study population formation. Matching of population controls conducted using exposure density sampling. Individuals excluded from cases were included in the population from which controls were sampled and could act as controls if they were as-yet-unexposed at the time of sampling.


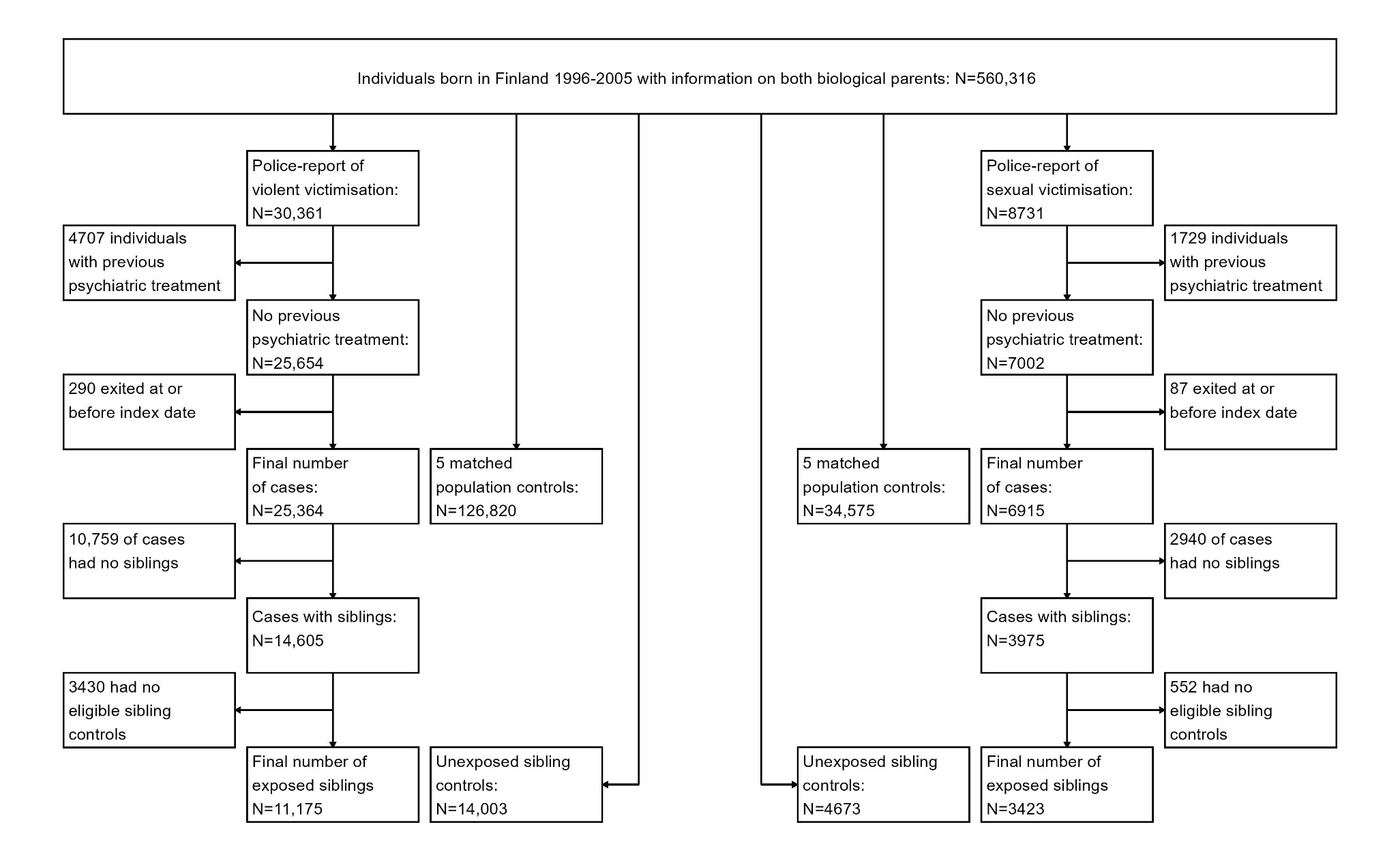


Figure S2: Results from the Schoenfeld residuals tests in the cohort comparisons. The models compare those exposed to victimisation with their five matched population controls (matched by sex, birth year, birth month and region of residence). The models are stratified by the matching variables and adjusted for two-parent family, parental education, foreign background and parental means-tested social assistance at birth and parental psychiatric disorders and violent and sexual crime convictions before birth.






Figure S3: Results from the Schoenfeld residuals tests in the sibling comparisons. The models compare exposed individuals to their unexposed siblings. The models are stratified by the sibling identifier and adjusted for sex, birth year, parental social assistance receipt and two-parent family at birth.

Figure S4: The associations between police-report of violent and sexual victimisation and anxiety disorders from the main analysis and a sensitivity analysis excluding stress-related disorders from the outcome. The population, unadjusted models compare those exposed to victimisation with their five matched population controls (matched by sex, birth year, birth month and region of residence). The model is stratified by the matching variables. The population, adjusted models are further adjusted for two-parent family, parental education, foreign background and parental means-tested social assistance at birth and parental psychiatric disorders and violent and sexual crime convictions before birth. The sibling comparison models compare exposed individuals with their unexposed siblings. The sibling models are stratified by the sibling identifier and adjusted for sex, birth year, parental social assistance receipt and two-parent family at birth. In the sensitivity analysis, individuals whose first anxiety disorder was a stress-related disorder were censored at the outcome date.


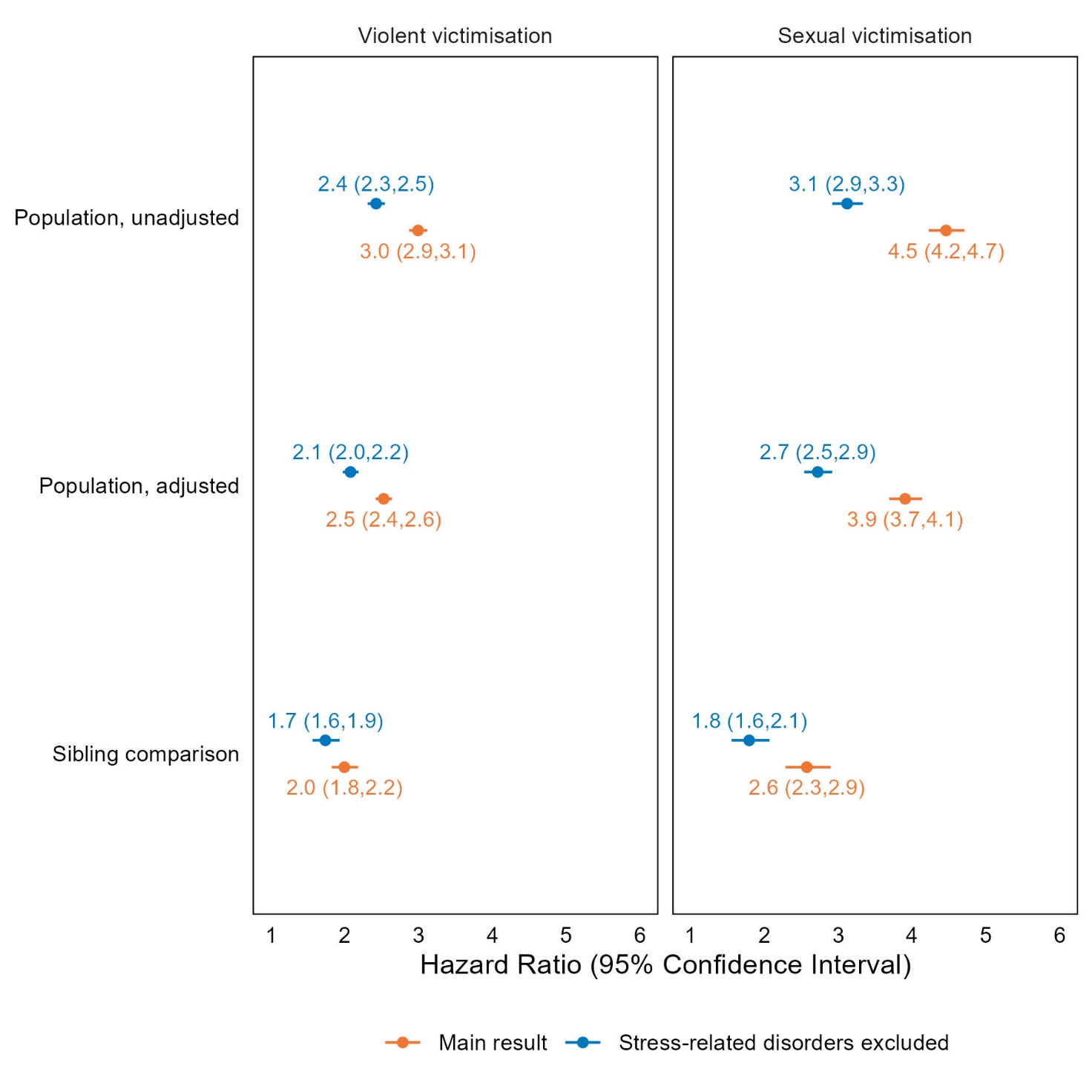

Supplement: Supplementary file 1 — Table S1. STROBE checklist. Figure S1. Flowchart of the study population formation. Figure S2. Results from the Schoenfeld residuals tests in the cohort comparisons. Figure S3. Results from the Schoenfeld residuals tests in the sibling comparisons. Figure S4. Results from a sensitivity analysis excluding stress‐related disorders from anxiety disorders. [file JCPP-67-1383-s001.docx]
